# Trauma outcomes in elderly patients referring to the emergency department via the rapid emergency medicine score, injury severity score, and geriatric trauma outcome score indices

**DOI:** 10.1186/s12245-025-01046-4

**Published:** 2025-10-30

**Authors:** Farzad Bozorgi, Touraj Assadi, Mohammad Sazgar, Fatemeh Jahanian, Mohammadreza Abdollahifar, Hamed Aminiahidashti

**Affiliations:** 1 Department of Emergency Medicine, Mazandaran University of Medical Sciences, Sari, Iran; 2 Orthopedic Research Center, Mazandaran University of Medical Sciences, Sari, Iran; 3 Diabetes Research Center, Mazandaran University of Medical Sciences, Sari, Iran; 4 School of Medicine, Mazandaran University of Medical Sciences, Sari, Iran; 5 Emam Khomeini Hospital, Amirmazandarani Boulevard, Sari, Mazandaran Province Iran

**Keywords:** Geriatric trauma, Rapid emergency medicine score, Injury severity score, Geriatric trauma outcome score

## Abstract

**Background:**

Accurate early risk stratification of older trauma patients is essential for appropriate triage and resource allocation. We compared the discriminatory performance of three prognostic scores—Rapid Emergency Medicine Score (REMS), Injury Severity Score (ISS), and Glasgow Coma Scale-based Trauma Outcome Score (GTOS)—for predicting in-hospital mortality in trauma patients aged 65 years and older.

**Methods:**

A single-center, retrospective cohort study was conducted, including all trauma patients aged 65 years or older admitted to the Emergency Department of Imam Khomeini Hospital (Sari, Iran) between September 2019 and March 2023 (*n* = 296). REMS, ISS, and GTOS were computed from registry and chart data. Discrimination was assessed using the area under the receiver operating characteristic curve (AUC-ROC) and compared with DeLong’s test. Multivariable logistic regression models were fit to estimate adjusted associations and predictive performance. Pre-specified subgroup analyses included patients ≥ 75 years and those with ISS ≥ 9.

**Results:**

This study examined 296 patients with trauma. Overall, in-hospital mortality was 6.0% (18/296). AUCs for predicting in-hospital mortality were: REMS 0.949 (95% CI 0.898–1.00), GTOS 0.928 (95% CI 0.881–0.949), and ISS 0.860 (95% CI 0.770–0.949). The overall difference in discrimination across scores was statistically significant (*p* = 0.031). In multivariable analysis, each one-point increase in REMS was associated with higher odds of in-hospital death (OR 1.51; 95% CI 1.45–1.58). Observed in-hospital mortality by REMS strata was: <8 = 1.2%, 8–10 = 8.3%, and ≥ 11 = 70.6%. GTOS maintained superior performance in subgroup analyses than REMS, including patients aged 75 years or older and those with an ISS of 9 or higher.

**Conclusion:**

In this cohort of older trauma patients, REMS demonstrated superior discriminatory ability for in-hospital mortality compared with GTOS and ISS, and may serve as a rapid, bedside tool to identify high-risk older trauma patients in the emergency department. External validation in larger, multi-center cohorts is recommended before broad implementation.

## Introduction

Individuals aged 65 years and older are classified as elderly [1]. In Iran, the proportion of the population aged 65 and above has been steadily increasing. According to the World Bank, in 2023, approximately 7.92% of Iran’s total population was aged 65 or older [2]. This demographic shift is attributed to factors such as increased life expectancy and declining fertility rate. Consequently, the elderly population is projected to comprise about 27.9% of Iran’s total population by 2050 [3].

In China, the growth rate of the elderly population has surpassed that of other age groups. By 2025, the percentage of the population over 60 years old is expected to constitute 12.3% of the total population [4], with this figure projected to rise further over the next 20 years [3]. Trauma is the fifth leading cause of death across all age groups, the fourth leading cause of mortality among individuals aged 55–64 years, and the ninth leading cause of death in individuals over 65 years of age [5, 6].

Age-related anatomical changes—such as reduced muscle mass and strength, decreased bone density, and limited joint flexibility—combined with physiological alterations like impaired vision and hearing, slower reflexes, reduced balance, and cognitive decline, make the management of elderly patients particularly challenging [7].

Trauma in elderly people typically culminate in worse clinical outcomes than in younger patients [6]. The mortality rate due to trauma considerably increases after the age of 65, with patients aged 35–44 having a mortality rate of 3.35%, whereas this rate is 6.66% for patients aged 75–84. Even if injury-induced trauma is not severe or immediately life-threatening, elderly patients who experience trauma exhibit an increased mortality rate within the subsequent 12 months [8]. Given these findings, accurate risk assessment for trauma in elderly people are critically important in trauma centers [9].

One of the innovative tools in this area is the Geriatric Trauma Outcome Score (GTOS), developed to provide a simple yet accurate method for predicting mortality in elderly trauma patients. GTOS is calculated using three key parameters: patient age, Injury Severity Score (ISS), and the need for blood transfusion within the first 24 h of admission [10]. Another well-known tool in this area is the Rapid Emergency Medicine Score (REMS), which is designed for the rapid assessment of patients’ clinical status in the emergency department to help medical teams identify high-risk patients and implement appropriate interventions [11].

Although trauma outcomes in elderly patients have been studied, most research targets younger or general trauma groups. Few studies directly compare scoring systems like REMS, ISS, and GTOS in older adults. This gap limits understanding of which tool best predicts risk and outcomes in this vulnerable population [12]. Accurate scoring systems offer key clinical benefits for elderly trauma patients. They help clinicians quickly identify high-risk cases, prioritize care, and allocate resources efficiently. These tools also predict outcomes, guide post-trauma planning, and support evidence-based decisions to improve survival and recovery [13, 14].

The collection and analysis of data concerning trauma outcomes in elderly people are of paramount importance, particularly when new metrics are utilized for accurate patient triage, considering the likelihood of severe injury, even in low-energy trauma, as well as the altered physiology in this population. With the growing elderly population and higher trauma-related mortality in this group, evaluating and comparing existing scoring systems is crucial. Identifying the most reliable tool can enhance clinical management, optimize resource use, and improve patient care in trauma centers.

Additionally, this information can help clinicians determine appropriate treatment strategies, allocate resources effectively despite existing limitations, and implement supportive measures based on evidence-based decision-making. Moreover, documenting incidents and injuries provides us with valuable information for monitoring and evaluating the healthcare system. This study is designed based on the hypothesis that the predictive performance of GTOS, REMS, and ISS differs in elderly trauma patients. Therefore, this study aims to assess trauma outcomes in elderly patients using REMS, ISS, and GTOS and to compare the predictive performance of these three scoring systems.

## Method

### Design and setting

This retrospective cohort study examined all trauma patients aged 65 years or older who were admitted to the emergency department of Imam Khomeini Hospital in Sari, Iran, between September 2019 and March 2023. This hospital is recognized as a Level 1 trauma center, which in our country represents the highest level of trauma care, providing full capacity for management of severe and complex trauma cases, including 24-hour availability of specialized surgical teams and intensive care facilities.

### Participants

All trauma patients aged ≥ 65 years were included using a total enumeration method. The study encompassed all elderly individuals presenting with trauma to the Emergency Department of Imam Khomeini Hospital, Sari, between September 2019 and March 2023, meeting the inclusion and exclusion criteria. Because the entire accessible population was studied, no formal sample size or power calculation was performed. This approach, common in observational research with finite populations, maximizes statistical power to detect clinically meaningful differences [15, 16].

### Data collection

Data were extracted from the systematic trauma registry at Imam Khomeini Hospital, Sari, and from patients’ medical records using a standardized checklist. Exclusion criteria included incomplete records, death upon admission, severe or isolated burns, and unknown injury mechanisms. Experienced emergency physicians assigned injury scores for each anatomical region based on clinical data and imaging, following ICD-10 guidelines. ICD-10 codes were converted into injury scores using the *International Classification of Diseases Programs for Injury Categorization in R (ICDPIC-R)* version 0.0.1, and their accuracy was verified by a trauma-trained emergency specialist. The software, developed from the U.S. National Trauma Data Bank (NTDB) and endorsed by the American College of Surgeons and AHRQ, ensured standardized injury classification.

### Statistical analysis

The data were analyzed using STATA version 17. After quality checks, descriptive statistics (frequencies, central tendency, and dispersion) were calculated. Data normality was assessed through histograms, Q-Q plots, and the Shapiro–Wilk test. Depending on variable types, the chi-square, Fisher’s exact, and Mann–Whitney U tests were used.

The chi-square test for trend evaluated whether mortality increased with higher REMS scores. To compare the accuracy of GTOS, ISS, and REMS in identifying risk of mortality among patients, the area under the ROC curve (AUC-ROC) was calculated. Covariate-adjusted ROC curves were constructed using multivariate logistic regression models that included each index and relevant confounders, with in-hospital mortality as the outcome. Adjusted AUCs were then estimated. Confounding variables were identified based on both theoretical and empirical considerations. Confounders were defined as variables related to both the predictor (REMS, GTOS, ISS) and outcome (Mortality) that could bias results.

The DeLong test compared AUCs between correlated indices [17]. Sensitivity analyses were conducted to test robustness, comparing REMS with GTOS and ISS separately, avoiding direct GTOS–ISS comparison to reduce bias. A 5% significance level was used. Patients were categorized into mortality and survival groups and compared via chi-square tests. Mortality prediction parameters (GTOS, TRISS, and REMS) were calculated for all participants, and AUC percentages were compared. Analyses were also repeated for subgroups: patients over 75 years and those with ISS > 9, representing severe injury. Multivariate logistic regression was further applied to evaluate REMS parameters.

### Ethical considerations

Accordingly, all data obtained from this study will be kept strictly confidential and will only be presented in the final report, ensuring that no individual has access to the data. Moreover, the study process and its objectives were explained to the study group. Participants were included only if they provided full and informed consent by completing and signing the relevant consent form. This project commenced after receiving approval from the Medical Ethics Committee (code: IR.MAZUMS.IMAMHOSPITAL.REC.1402.145). All principles of medical ethics and the Declaration of Helsinki were followed throughout the research. The principles of confidentiality and no imposition of costs were also observed.

## Results

### Sample characteristics

This study examined 296 trauma patients aged 65 years or older who were admitted to the emergency department of Imam Khomeini Hospital in Sari, Iran, between September 2019 and March 2023. Among these, 198 (66.89%) were male, and 98 (33.11%) were female. The majority of cases were related to traffic accidents, accounting for 156 patients (52.70%), followed by falls from height, with 62 cases (20.80%), falls on the same level, with 46 cases (15.54%), and the remaining 32 cases involved other types of traumas, including penetrating trauma and assault. Forty-three patients (14.52%) required packed red blood cell (RBC) transfusions within the first 24 h of admission. The mortality rate was 6% (*n* = 18).

### Mortality-stratified characteristics

A total of 296 patients were included (278 survivors, 18 deceased). Deceased patients were older (77.1 ± 1.7 vs. 73.8 ± 7.6 years; *p* = 0.072) and had higher ISS, GTOS, and REMS scores but lower GCS (*p* < 0.001). They also showed higher systolic/diastolic blood pressure and heart rate (all *p* < 0.05), lower oxygen saturation, and significant differences in several AIS domains, particularly chest injuries (*p* = 0.008). Blood transfusion was less frequent among deceased patients (44.4% vs. 88.1%; *p* < 0.001) (Table 1). Patients with REMSs less than 8, between 8 and 10, and greater than 11 had mortality rates of 1.2%, 8.3%, and 70.58%, respectively (*p* < 0.001). A 1-point increase in the REMS score was associated with an odds ratio of 1.51 for in-hospital death (95% confidence interval [CI]: 1.45–1.58).

**Table 1:** Comparison of epidemiological and clinical findings between deceased patients and surviving patients

Variable	Category	Survival Group	Mortality Group	*P* Value
Gender, n (%)	Male	184 (66.18)	14 (77.77)	> 0.001
	Female	94 (33.82)	4 (23.23)	
Age, Mean ± SD		73.77 ± 7.65	77.11 ± 1.69	0.072
ISS, Median (Q1-Q3)		7 (4–10)	20.5 (9–32)	> 0.001
Blood transfusion, n (%)	Yes	245 (88.12)	8 (44.44)	> 0.001
	No	33 (11.88)	10 (55.56)	> 0.001
Blood unit number, Mean ± SD		1.12 ± 0.33	1.25 ± 3.8	> 0.001
AIS of head and neck, Median (Q1-Q3)		2 (1–2)	3 (3–4)	> 0.001
AIS ≤ 3		82 (90.11)	9 (9.89)	> 0.001
AIS of face, Median (Q1-Q3)		1 (1–2)	1.5 (1–2)	0.819
AIS ≤ 3		50 (92.59)	4 (7.41)	> 0.001
AIS of chest, Median (Q1-Q3)		2 (1–2)	2 (1–3)	0.249
AIS ≤ 3		84 (92.31)	6 (57.00)	0.008
AIS of abdomen, Median (Q1-Q3)		2 (1–2)	1 (1–2)	0.348
AIS ≤ 3		51 (89.47)	6 (10.53)	> 0.001
AIS of extremities, Median (Q1-Q3)		2 (2–3)	4 (3–4)	> 0.001
AIS ≤ 3		182 (97.33)	5 (2.67)	> 0.001
Systolic blood pressure (mmHg), Mean ± SD		131.88 ± 21.84	146.67 ± 38.88	0.009
Diastolic blood pressure (mmHg), Mean ± SD		80.99 ± 11.38	87.28 ± 21.15	0.034
Heart rate, Mean ± SD		83.56 ± 10.64	93.44 ± 31.21	0.002
Respiratory rate, Mean ± SD		16.31 ± 2.55	17.22 ± 4.56	0.165
Oxygen saturation (%), Mean ± SD		97.59 ± 1.39	94.78 ± 4.91	> 0.001
GCS, Mean ± SD		14.64 ± 1.47	9.17 ± 2.36	> 0.001
Mechanism	Penetrating trauma	10 (100)	0 (0)	> 0.001
	Blunt trauma	5 (55.56)	4 (44.44)	
	Road traffic accidents	145 (92.95)	11 (7.05)	
	Fall from height	60 (96.77)	2 (3.23)	
	Ground-level fall	45 (97.83)	1 (2.17)	
	Assault / Interpersonal violence	13 (100)	0 (0)	
REMS, Mean ± SD		6.08 ± 1.29	11.11 ± 2.54	> 0.001
ISS, Median (Q1-Q3)		7 (4–10)	20.5 (9–32)	> 0.001
GTOS, Mean ± SD		93.46 (17.57)	143.22 (32.34)	> 0.001
REMS, n (%)	5	102	0	> 0.001
	6–7	138	3	
	8–10	33	3	
	11–12	4	6	
	13 and higher	1	6	

ISS: Injury Severity Score; AIS: Abbreviated Injury Scale; GCS: Glasgow Coma Scale; REMS: Rapid Emergency Medicine Score; GTOS: Geriatric Trauma Outcome Score

### Comparison of predictive accuracy using AUC-ROC

**Fig. 1:**
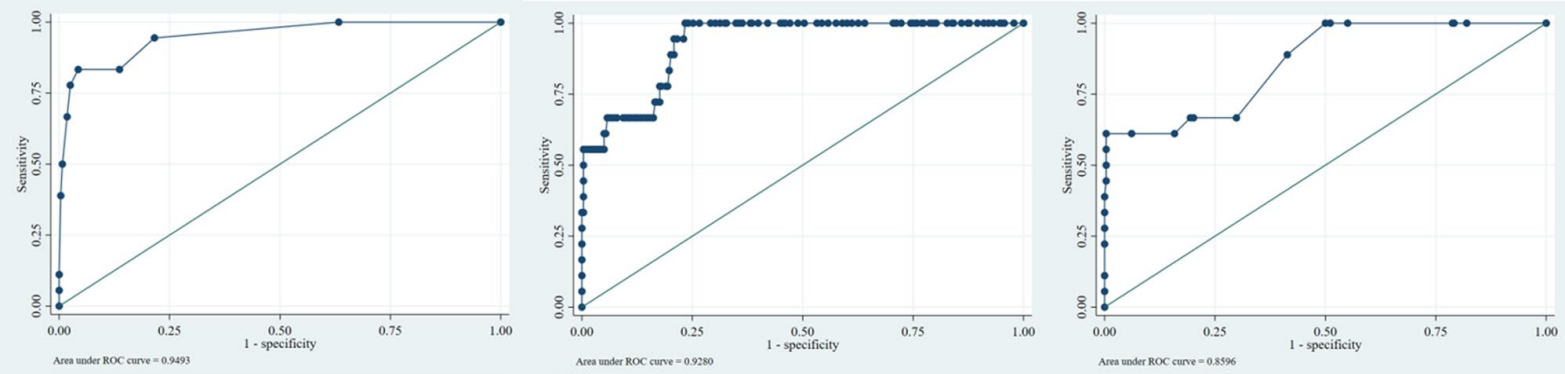
Comparison of ROC curves for REMS, GTOS, and ISS indices in the investigated population (respectively from left to right)

The accuracy of the REMS, ISS, and GTOS values in predicting patient mortality was compared via the AUC-ROC curve. The results revealed that the AUC was 0.928 for the GTOS (95% CI: 0.881–0.949), 0.949 for the REMS (95% CI: 0.898–1.00), and 0.860 for the ISS (95% CI: 0.770–0.949). The difference between the three indices was statistically significant according to DeLong’s test (*p* = 0.031). (Fig. 1).

### Comparison of predictive accuracy using adjusted AUC-ROC

**Fig. 2:**
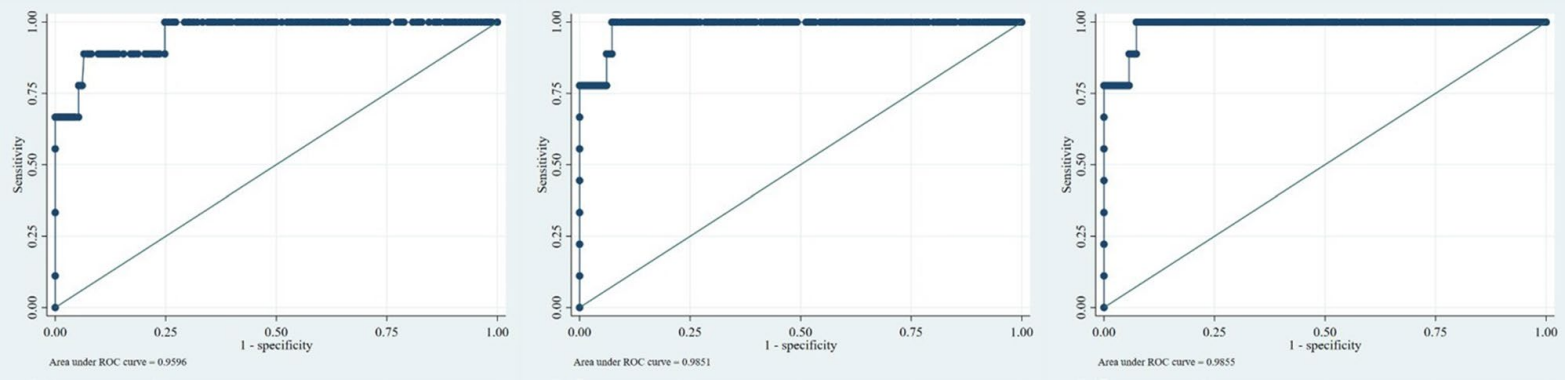
Comparison of adjusted ROC curves for REMS, GTOS, and ISS indices in the investigated population (respectively from left to right)

Multivariate logistic regression models were constructed separately for each predictive index using their respective confounders. The covariate-adjusted AUC for REMS (adjusted for age, SBP, DBP, mechanism of injury, PR, GCS, Blood unit number, and Oxygen saturation) was 0.960 (95% CI: 0.905–1.00). For GTOS (adjusted for age, GCS, Blood unit number and Oxygen saturation, and mechanism), the adjusted AUC was 0.985 (95% CI: 0.965–1.00), and for ISS (adjusted for age, GCS, SBP, Blood unit number and Oxygen saturation, and mechanism), the adjusted AUC was 0.986 (95% CI: 0.965–1.00).(Fig. 2).

### Subgroup analysis

The number of patients aged 75 years or older was 140. The survival and mortality groups consisted of 126 and 14 patients, respectively. The mortality rate was calculated to be 10%. The AUC was 0.826 for the GTOS (95% CI: 0.728–0.924), 0.927 for the REMS (95% CI: 0.834–1.00), and 0.815 for the ISS (95% CI: 0.705–0.924). The difference between the three indices was not statistically significant according to DeLong’s test (*p* = 0.211).

An ISS ≥ 9 is considered severe injury, and the total number of patients in this category was 104. The numbers of these patients in the survival and mortality groups were 88 and 16, respectively, resulting in a mortality rate of 15.38%. The AUC was 0.941 for the GTOS (95% CI: 0.859–1.00), 0.937 for the REMS (95% CI: 0.862–1.00), and 0.947 for the ISS (95% CI: 0.851–1.00). DeLong’s test indicated no statistically significant difference between these AUCs (*p* = 0.907).

### Sensitivity analysis

The results showed that the AUC for REMS was slightly higher than that for GTOS, but the difference was not statistically significant (*p* = 0.468). Similarly, REMS versus ISS showed an AUC of 0.95 versus 0.86, confirming the robustness of REMS as the most predictive index (*p* = 0.073).

## Discussion

### Summary of main findings

We compared GTOS, REMS, and ISS for mortality prediction. REMS had the highest AUC (0.949) versus GTOS 0.928 (and ISS) 0.860. (The difference between the three indices was statistically significant (*p* = 0.031). Although the difference in AUCs between REMS, GTOS, and ISS reached statistical significance, the absolute magnitudes of these differences are relatively small and warrant cautious interpretation regarding clinical relevance.

Although the three indices differed significantly (*p* = 0.031), the absolute AUC differences were small and warrant cautious interpretation. A 0.02–0.03 change in AUC may not affect bedside decisions unless it meaningfully alters sensitivity or specificity at clinical cut-points. Thus, tool selection should consider not only discrimination but also calibration, the sensitivity/specificity tradeoff at chosen thresholds, ease and speed of use, and the downstream consequences of false positives versus false negatives (e.g., unnecessary resource use vs. missed high-risk patients) [18–20].

After multivariate adjustment, predictive accuracy improved for all indices—especially GTOS and ISS, which reached adjusted AUCs of 0.985 and 0.986—indicating that including age, blood pressure, transfused units, consciousness level, and injury mechanism markedly enhances prediction.

Subgroup analyses showed that REMS had the highest discrimination in patients ≥ 75 years (AUC = 0.927) but differences among indices were not statistically significant (*p* = 0.211). In those with severe injuries (ISS ≥ 9), ISS and GTOS demonstrated the highest AUCs (0.947 and 0.941, respectively) versus REMS (0.937), again without significant differences (*p* = 0.907). Sensitivity analyses indicated REMS was the most consistent performer in the overall cohort, though didn’t reach statistical differences compared to other indices were limited (*p* = 0.073–0.468). Overall, all three indices show substantial prognostic utility for in-hospital mortality; REMS appears robust in adjusted models, but these findings require cautious interpretation and external validation.

Additionally, factors such as mechanism of injury and blood transfusion volume during hospitalization are independent predictors of increased mortality in trauma patients. Also, Vital signs, including blood pressure, heart rate, oxygen saturation, and GCS score, differed significantly between deceased individuals and survivors.

### Comparison with previous studies

Numerous studies have demonstrated that the risk of mortality increases up to fivefold with a systolic blood pressure of less than 90 mmHg [21]. In elderly trauma patients who are on antihypertensive medications, a blood pressure below 110 mmHg has been considered a cutoff score for predicting massive blood transfusion [22]. Although relying merely on vital sign assessments is insufficient to guide the management of elderly trauma patients, hemodynamic stability is crucial for appropriate resuscitation [23].

Some studies have shown that individuals over 75 years of age have a higher mortality rate than those between 65 and 74 years of age [24]. Similarly, in our study, a non-significant difference was found between mortality rates and increasing age. Also, the GCS score, and oxygen saturation were the only REMS parameters strongly correlated with mortality (26). Furthermore, the REMS is a robust predictor of in-hospital mortality in polytrauma patients [25]. Our study suggests that the REMS is a strong predictor of mortality in elderly trauma patients. These results align with a recent systematic review and meta-analysis demonstrating REMS’s variable but often strong discrimination for in-hospital mortality across ED settings (AUC range 0.52–0.986), with high predictive performance reported in roughly two-thirds of studies. The concordance between our findings and the broader literature supports REMS’s robustness and external validity. The improved accuracy after covariate adjustment underscores the value of incorporating age, blood pressure, and consciousness level into risk models. Overall, REMS is a simple, effective tool for early ED mortality risk stratification and triage [26].

While heart rate and respiratory rate alone are not predictive of mortality, the other four REMS parameters are, with the GCS being the most prominent. Some studies have also demonstrated that the REMS is a suitable tool for predicting mortality rates in trauma patients, particularly those with traumatic brain injury [27]. As shown by our research findings, the REMS better predicts mortality than the ISS and the GTOS do in elderly trauma patients. Huang et al. reported strong discrimination for GTOS (AUC = 0.917) with high sensitivity and specificity, supporting its utility beyond geriatric cohorts. In our cohort, however, REMS outperformed both GTOS and ISS. This likely reflects REMS’s inclusion of dynamic physiological variables—blood pressure, respiratory rate, and level of consciousness—that more directly capture acute physiological deterioration than GTOS’s age- and transfusion-based components. Thus, REMS may better integrate physiological response with anatomical injury, making it a more robust mortality predictor across diverse trauma populations [28].

The GTOS serves as a reliable and easily calculable measure that can assist in determining the likelihood of mortality for elderly patients following trauma. Additionally, the GTOS can be employed to establish care goals with patients [29]. Studies comparing the GTOS and ISS in elderly patients have also demonstrated that the GTOS has superior performance over the ISS in mortality prediction [30]. While the GTOS appears to predict mortality in elderly trauma patients with considerable accuracy, this score largely depends on the ISS criteria, which are known for their subjectivity and suboptimal reliability [31]. In our study, the results also demonstrated a greater AUC for the GTOS (0.928) than for the ISS (0.860). The improved performance of the GTOS in predicting mortality among elderly trauma patients may be attributed to the inclusion of physiological parameters. Age and posttraumatic blood transfusion are associated with morbidity and mortality during hospitalization in elderly trauma patients, which helps the GTOS assess patients’ condition more comprehensively. The GTOS is superior to the TRISS, a composite scale involving the ISS, GCS, SBP, and respiratory rate, in predicting mortality in trauma patients over 65 years of age [32]. In contrast, some studies have suggested that the GTOS does not function as well as the TRISS in predicting mortality in elderly trauma patients [33, 34]. This may be because TRISS includes both anatomical and physiological parameters, such as the Glasgow Coma Scale and blood pressure, which provide a more dynamic picture of the patient’s condition. In contrast, GTOS relies only on age, ISS, and early blood transfusion, which may not fully capture the physiological instability and comorbidities common in older adults. Moreover, differences in study design, patient characteristics, and healthcare systems may also explain variations in predictive performance. Despite these factors, our study found that GTOS still demonstrated good predictive ability, indicating its practical value in mortality prediction for elderly trauma patients.

The prediction of mortality by both the ISS and the GTOS yielded AUCs of 0.66 for the ISS (95% CI: 0.59–0.74) and 0.68 for the GTOS (95% CI: 0.61–0.76). The optimal cutoff points were ≤ 28 for the ISS and ≤ 142 for the GTOS [35]. The requirement for a transfusion history within the first 24 h may delay the accurate calculation of predictions. On the other hand, the GTOS offers the advantage of rapid and straightforward scoring. Therefore, as a “nascent” trauma scoring system, further studies are necessary to confirm the value of the GTOS in the context of trauma among older adults.

Our findings have important clinical implications. First, REMS provides a rapid and reliable tool for early risk stratification of trauma patients in the emergency setting, enabling timely interventions and prioritization of care. Second, GTOS is particularly valuable for predicting mortality in elderly patients, providing a simple and practical measure to support individualized care planning, goal setting, and the allocation of medical resources. Together, these scoring systems can help clinicians make informed decisions, optimize patient management, and improve outcomes across diverse trauma populations.

### Limitations

We acknowledge several limitations that affect interpretation and generalizability. First, the single-center, Level-I setting in northern Iran limits external validity; the very high adjusted AUCs reported may not hold without external validation. Second, the small number of events (overall *n* = 18; e.g., 14 deaths in the ≥ 75 subgroup, 16 in the ISS ≥ 9 subgroup) raises concerns about model stability and overfitting—multivariable models with low events-per-variable can yield over-optimistic discrimination; future studies should use larger event counts or penalized regression and rigorous internal validation (bootstrapping or cross-validation). Third, selection and information biases are possible: excluding patients with incomplete records and those dead on arrival may skew the sample toward less severe cases, and the complete-case approach risks bias if missingness is not random; registry variables also depend on documentation quality. Fourth, unmeasured confounding likely remains (frailty, baseline function, anticoagulant use, comorbidity severity, prehospital care and timing), which could influence index performance—future work should incorporate frailty measures. Finally, the study period (Sept 2019–Mar 2023) may include temporal changes in practice (including pandemic effects), and prehospital deaths were not captured, further limiting generalizability.

## Conclusion

In this retrospective cohort of 296 trauma patients aged ≥ 65 years, REMS showed superior predictive accuracy for in-hospital mortality compared with GTOS and ISS (AUC 0.949 vs. 0.928 and 0.860; *p* = 0.031). Each one-point increase in REMS raised mortality odds by 51% (OR 1.51, 95% CI 1.45–1.58). Patients with REMS ≥ 11 had a 70.6% mortality rate, highlighting its clinical utility for early triage. Despite these strengths, the single-center design and small number of deaths limit generalizability, warranting confirmation in larger, multicenter studies.

## Data Availability

No datasets were generated or analysed during the current study.
